# Predicting Child Protective Services (CPS) Involvement among Low-Income U.S. Families with Young Children Receiving Nutritional Assistance

**DOI:** 10.3390/ijerph14101197

**Published:** 2017-10-11

**Authors:** Kristen S. Slack, Sarah Font, Kathryn Maguire-Jack, Lawrence M. Berger

**Affiliations:** 1 School of Social Work, University of Wisconsin-Madison, 1350 University Avenue, Madison, WI 53706, USA; lmberger@wisc.edu; 2 Institute for Research on Poverty, University of Wisconsin-Madison, 1180 Observatory Drive, Madison, WI 53706, USA; 3 Department of Sociology and Criminology, The Pennsylvania State University, 505 Oswald Tower, University Park, PA 16802, USA; saf252@psu.edu; 4 The Ohio State University College of Social Work, 1947 N College Rd, Columbus, OH 43210, USA; maguirejack.1@osu.edu

**Keywords:** child protection, child maltreatment, safety net, poverty, welfare

## Abstract

This exploratory study examines combinations of income-tested welfare benefits and earnings, as they relate to the likelihood of child maltreatment investigations among low-income families with young children participating in a nutritional assistance program in one U.S. state (Wisconsin). Using a sample of 1065 parents who received the Special Supplemental Nutrition Assistance Program for Women, Infants, and Children (WIC) benefits in late 2010 and early 2011, we find that relying on either work in the absence of other means-tested welfare benefits, or a combination of work and welfare benefits, reduces the likelihood of CPS involvement compared to parents who rely on welfare benefits in the absence of work. Additionally, we find that housing instability increases the risk of CPS involvement in this population. The findings from this investigation may be useful to programs serving low-income families with young children, as they attempt to identify safety net resources for their clientele.

## 1. Introduction

Many decades of child maltreatment research have yielded information on the etiology of child maltreatment or child protective services (CPS) involvement in the United States [1,2,3,4,5,6,7,8]. These studies, in addition to studies conducted in other developed countries, have repeatedly pointed to links between various indicators of poverty and maltreatment-related indicators [1,2,3]. Many of these analyses are based on low-income samples, in part because poverty is a commonly identified risk factor for child maltreatment and in part because child maltreatment, including its various proxy measures, is a relatively rare event at the population level [1,2,7,8]. In the United States, children in low-income families are significantly more likely to experience every form of child maltreatment than their higher income counterparts [9]. This association is strongest for child neglect, and weakest for sexual abuse [9]. Since most low-income parents do not maltreat their children [8], studying “within-group” variation in CPS involvement among low-income families provides an opportunity for identifying risk and protective factors that drive the correlation between poverty and child maltreatment.

Identifying samples that are representative of low-income families can, however, still be challenging. In the United States, the economic safety net is comprised of various income-tested programs, each with their own eligibility requirements and application procedures. Families that attempt to navigate this complex service array elect to participate in different combinations of programs if they can meet the necessary requirements [10]. As such, there is no clear “package” of welfare benefits common across all, or even most, low-income families, making it difficult to identify appropriate samples for studying within-group CPS risk among families in poverty. Potential focal populations are families receiving benefits from means-tested programs, such as Temporary Assistance for Needy Families (TANF), a cash assistance program largely hinged on employment activities, or the Supplemental Nutrition Assistance Program (SNAP), which provides monthly benefits for purchasing household food. Extensive prior research has been conducted on the predictors of CPS involvement among TANF recipients, particularly around the time of major United States welfare reforms in the mid-1990s [11,12,13,14,15,16,17,18,19]. Since then, TANF participation rates have declined by nearly 70% [20], and rates of participation among eligible families are now less than 25% [21]. Participation rates in SNAP are much higher, with 83% of those eligible receiving benefits nationally [22]. However, because SNAP is not targeted specifically toward families with children, the SNAP recipient population is heterogeneous with respect to the presence and ages of children in the household, and thus may not adequately capture low-income families at risk of child maltreatment.

Few child maltreatment studies have considered the population of families receiving benefits from the the Special Supplemental Nutrition Assistance Program for Women, Infants, and Children (WIC) Program. WIC is a federally-funded nutrition assistance program for pregnant women and families with children under the age of five, from households with incomes below 185% of the federal poverty line [23]. In most states, the benefits are provided in the form of vouchers that are redeemable for specific food items at authorized retailers. The program also provides nutrition education and counseling at WIC clinics, as well as screenings and referrals to other health and social services. Within the literature examining the impact of public benefits on child maltreatment, only one study has examined the associations between the receipt of WIC and substantiated child abuse or neglect [24]. The authors found that families receiving either WIC or SNAP (or both) were less likely to have a substantiated child abuse or neglect investigation [24]. This suggests that the WIC program may have a protective influence on CPS risk, a point to which we return in the discussion section.

### Current Study

In Wisconsin, where the present study is based, just over half of eligible families (56%) participate in WIC, which is slightly less than the national participation rate of 60.2% [25]. Thus, although WIC participants in Wisconsin do not reflect all caregivers with young children from households under 185% of the federal poverty line, they reflect a majority of this population. WIC is provided to low-income parents of young children, an age group at the greatest risk of child maltreatment [26]. Studying the extent to which WIC-receiving families become involved with CPS and the correlates of that involvement can further our understanding of the phenomenon of child maltreatment among low-income families. It extends the research on the correlates of CPS risk within low-income populations by focusing on a largely overlooked subgroup, those participating in a popular means-tested nutritional assistance program in the United States (WIC).

## 2. Methods

### 2.1. Study Design

For the present study, we used data from the Family Support Study (FSS), a survey of Women, Infant, and Children (WIC) recipients across the state of Wisconsin. The FSS was linked with longitudinal state administrative data that include child welfare involvement, public benefits receipt, child support payments, and earnings. FSS survey data were collected during the last two months of 2010 and the first two months of 2011. A total of 22 WIC offices from across the state participated in the study, as well as WIC recipients participating in a home visiting program in the city of Milwaukee. As WIC applicants were approved for benefits, or as WIC participants came into program offices for the re-certification of benefits, survey packets were distributed to individuals by program staff. Survey packets included an introductory letter describing the project as a study of how family characteristics and situations are related to the kinds of services and benefits that families receive. Families were informed that they were being asked to participate because they are currently receiving services from a program providing supports to families in Wisconsin. Also included in the packet was a human subjects consent form, the survey tool, and a stamped return envelope. Packets were distributed to the home visiting participants during scheduled home visits by program staff during the field period. All subjects gave their informed consent for inclusion before they participated in the study. The study protocol was approved by the University of Wisconsin-Madison Social Sciences Institutional Review Board (Project identification code: 2013-0884).

Survey items were integrated into a self-administered format, available in both English and Spanish. A total of 2192 survey packets were distributed across sites, and a total of 1087 surveys were returned (49.5%). Return ratios for the surveys (i.e., the number of surveys returned over the number of surveys distributed in each site) ranged from 1/3 to just over 2/3 (A true response rate was not available, since the denominator does not include clients who may have refused the survey packet). The Home Visiting Program site comprised 7% of the sample, and the return ratio for that site was low (33%), relative to the WIC sites. Of the returned surveys, 22 were excluded due to extensive missing data or because the respondent did not appear to be a current WIC recipient, for a total of 1065 completed surveys.

### 2.2. Data Sources and Measures

A primary data source for the study is the FSS survey, which involved various measures of parenting, social support, parental depression, and indicators of economic hardship, as well as family structure and sociodemographic variables known to be (positively or negatively) associated with the risk of either child maltreatment or involvement in the CPS system. Dichotomous measures indicated parents who were less than 25 years of age, had more than two minor age children in the home, had at least one child under the age of two, were married or living with a partner, had less than a high school degree (12 years of elementary and secondary education) or more than a high school degree (one or more years of post-secondary education, including trade school; reference category = high school degree only, i.e., 12 years of elementary and secondary education), and identified as non-Hispanic Black or Hispanic (reference category = non-Hispanic White or other racial or ethnic identities) (The small number (*n* = 58) of respondents who identified as another race or ethnicity reflected disparate characteristics. However, as a group, their association with the CPS outcome was most consistent with respondents who identified as non-Hispanic White). Analyses also controlled for housing instability (more than one housing move in the past year) and for belonging to the group of home visiting program participants, who may have differed in both measured and unmeasured ways from the respondents recruited from WIC Program offices.

Parenting stress was measured with an eight-item scale, consisting of items such as “My children seem to be ‘on my nerves’ most of the time”, “I often feel tired, worn out, or exhausted from raising my family”, and “I feel good about my parenting abilities” (reverse-coded). Social support is an 11-item scale, consisting of items such as “There are people in my life who encourage and support me in meeting my goals”, and “I do not know many people who I can talk to about my problems.” Depressive symptomatology was measured using the CES-D [27]. All three scale measures demonstrated strong internal reliability (i.e., Cronbach’s alpha > 0.85).

Data on income and benefit receipt and amounts in the FSS sample was derived from the 2010 Multi-System Person File (MSPF) longitudinal administrative database created and maintained by the Institute for Research on Poverty (IRP) at the University of Wisconsin-Madison. FSS sample members were linked to the MSPF by IRP programmers using identifying information (e.g., names, birthdates, children’s names). A total of 93% of FSS sample members could be linked to the MSPF database; 7% were not found in the MSPF database, presumably because they do not have a work history based on taxable earnings and have not been formally served by any of the systems included in the database (Although all FSS sample members received WIC benefits, WIC is not currently integrated in the MSPF. Thus, it is possible that a small percentage of our sample members have received WIC benefits in the absence of any other services or benefits from other systems currently included in the MSPF).

The MSPF provided information on the receipt and amounts of a range of income sources, including earnings from work, Temporary Assistance for Needy Families (TANF), Supplemental Nutrition Assistance Program (SNAP), child care subsidies, unemployment insurance (UI) benefits, Supplemental Security Income (SSI), Social Security Disability benefits (SSDI), and child support. A continuous variable approximating total income was constructed by summing the 2010 totals of each of these income sources. Additionally, four dichotomous variables were created that reflect combinations of earnings and welfare receipt. First, respondents with an average of at least $50 per month in combined TANF and SNAP benefits were coded as receiving “welfare” in 2010; respondents with an average of $50 per month in earnings were coded as having worked in 2010. The combinations of these two variables yielded four dichotomous measures reflecting No Work/No Welfare, Welfare only, Work only, and Work and Welfare, combined. An additional fifth dichotomous variable was constructed reflecting the receipt of any SSI or SSDI benefits for the primary caregiver or household children. This variable was included in analyses to control for chronic health conditions that may affect caregiving abilities and demands. 

The dependent variable for our analyses was having an investigated child maltreatment report in the 18 months following the survey interview. We focus on investigated reports (as opposed to substantiated reports) given prior research that has shown similar rates of re-reports to CPS for families with substantiated and unsubstantiated reports [28] and it is not clear that substantiation is a meaningful demarcation of maltreatment or risk [29,30,31]. In the FSS sample, 7.4% of respondents were involved in an investigated CPS report during the post-survey follow-up period. A control variable for investigated CPS reports prior to survey participation was also constructed. Given the observational nature of the study, controlling for prior CPS contacts helps reduce selection bias attributed to unmeasured characteristics that may be correlated with both future CPS involvement and our key predictor variables.

It is important to state that CPS involvement is only a proxy for child maltreatment, and is subject to error in both directions (under-identification and over-identification of child maltreatment). However, CPS involvement is associated with a range of problematic parenting behaviors and home environment conditions [5,32] and has the added advantage of shedding light on the cross-system involvement of low-income families.

### 2.3. Statistical Analysis

Chi-square tests (for dichotomous predictors) and one-way ANOVAs (for continuous scale variables and for income) were run to test for differences in sample characteristics across welfare and work categories. Logistic regression analysis was used to predict the binary outcome of CPS involvement. We pay most attention to the role of earnings and welfare benefits with respect to CPS risk. We control for total income so that the status or condition of working and/or receiving welfare benefits is the focus, as opposed to omitting a control for income and conflating these statuses with the varying income amounts associated with them. Several sensitivity tests were conducted using different versions of welfare and work indicators, including measures that incorporated additional income sources and cut-off amounts. The results were reasonably consistent across these different measurement strategies.

## 3. Results

Table 1 in the Results section displays the characteristics of the total sample, and by welfare and work combinations. Of the total sample (*n* = 1065), 19.7% fell into the No Work/No Welfare group (*n* = 210), 29.1% were in the Welfare only group (*n* = 310), 19.1% were in the Work only group (*n* = 203), and 32.1% were in the Work and Welfare group (*n* = 342).

Chi-square one-way ANOVA tests were conducted to determine whether there were statistically significant differences in the sample characteristics by welfare and work combinations. In terms of the key dependent variable of interest, 7.4% of the overall sample had new CPS involvement, and this rate varied across Work and Welfare categories, from 2 to 13%. In terms of prior CPS involvement, rates varied across Work and Welfare categories from 6 to 24%.

There were significant differences across groups for all sociodemographic characteristics. Parents in the No Work/No Welfare group were distinguished by four characteristics: they were most likely to be married or cohabiting and under 25 years old, to have more than two children in the home, and had the lowest individual income. In contrast, parents in the Welfare Only group were more likely than all other groups to be participants in the Home Visiting program and have less than a high school education. Parents in the Work only group were distinguished by three characteristics: they were most likely to have a post-secondary education and to have a child under the age of two, and least likely to have more than two children. Lastly, the Work and Welfare parents were least likely to be married or cohabiting, more likely to have housing instability, and had the highest individual income. The Welfare only group had the highest rate of SSI receipt compared to all other groups.

Respondents in the Welfare only and combined Welfare and Work groups had higher levels of depressive symptoms, and the Work only group reported a greater level of social support than the Welfare only group. There were no statistically significant differences across the welfare and work groups for the parenting stress measure.

### Logistic Regression Results

The results of a logistic regression predicting CPS involvement within 18 months of the survey are found in Table 2. Of key interest, we found that parents in the Work Only group and the Work and Welfare group were less likely to be investigated by CPS as compared with the Welfare Only reference group. The finding was marginally significant (*p* < 0.10) for the Work and Welfare group. In other iterations of the model (not shown), we substituted different reference groups. The Welfare only group (OR 4.34, *p* < 0.05) and the No Work/No Welfare group (OR 5.12, *p* < 0.10) had a greater risk of CPS involvement than the Work only group, and the Welfare only group was more likely (OR 1.84, *p* < 0.10) than the Welfare and Work group to have CPS involvement.

Housing instability and having a child under the age of two were associated with an increased risk of CPS involvement, whereas having any post-secondary education was associated with a marginally statistically significant decreased risk. We found no statistically significant associations of self-reported depressive symptoms, parenting stress, or social support with CPS involvement. Lastly, logged annual individual income was associated with an increased risk of CPS involvement. The Nagelkerke r-square statistic is 31.5%.

## 4. Discussion

Continuing to receive welfare in the absence of work has been repeatedly shown to elevate the risk of CPS involvement in low-income samples [5,18,33,34]. It is unclear whether this is because relying only on welfare benefits signals a greater level of economic disadvantage that directly or indirectly elevates risk, a greater visibility to potential child maltreatment reporters (i.e., surveillance bias—[35,36]), or a selection effect whereby unmeasured characteristics of the parent or family predict both welfare receipt and CPS risk. We attempt to address the relative economic disadvantage hypothesis by controlling for total respondent income, so that categories of work and welfare receipt more accurately reflect the conditions of working and/or receiving welfare rather than the income amounts associated with each status.

In the current study, we find that work in the absence of or in combination with welfare benefits operates as a protective influence on the risk of CPS involvement, independent of income levels. These findings may be driven by a greater time spent by children in day care settings [37], reduced stress from fewer caregiving hours or from better economic situations [38,39], or (for the Work only group) less surveillance by welfare benefit programs [35]. Working may also affect the human and social capital of WIC recipients in ways that improve caregiving dynamics in the home.

The finding that respondent income is associated with an increased risk of CPS involvement seems counterintuitive. In population-based samples, income is often shown to have an inverse relationship with child maltreatment outcomes [4,9], whereas in low-income samples, the evidence has been mixed [5,18,40,41,42]. One possible explanation for our finding is that the income measure used does not include information on spouse or partner income contributions. The No Work/No Welfare group has a higher rate of marriage/cohabitation (73.8%), particularly compared to the Welfare only (61.3%) and combined Welfare and Work (52.3%) groups, and a significantly lower annual income level than all other groups (<$1000). There are likely unmeasured contributions to income that are especially pronounced for this group, which controlling for cohabitation alone does not capture. The No Work/No Welfare group also has a greater percentage of families with more than two children compared to all other groups, again suggesting that respondents in this group may be more likely to rely on other, potentially informal, sources of income and support in order to provide stay-at-home care for children. Still, the objective of this study was to understand whether the various status combinations of work and welfare predict CPS involvement, net of the income amounts that these status combinations reflect. As such, we opt to control for income, despite its potential limitations in our data.

Net of work, welfare, and income, housing instability was positively associated with a CPS investigation. Housing-related hardships have been linked with child maltreatment and CPS involvement in a number of studies [43,44,45]. Although housing problems, such as instability, commonly stem from poverty or financial incapacity, our findings are consistent with prior research showing that housing instability constitutes a unique stressor that can lead to harsh and neglectful parenting, net of other financial hardships [45].

One of the considerations from this research is whether earnings and welfare are reliable sources of income in the sample. In the Work only group (*n* = 203), the majority (71.9%) had earnings in all four quarters of 2010. Sixty-eight percent of the Welfare only group (*n* = 310) received SNAP benefits in 11 or 12 of the months in 2010, while only 26% received TANF benefits in one or more months of the year (with most receiving this benefit for fewer than six months). Among those who combined work and welfare (*n* = 342) in 2010, 57.9% had earnings in all four quarters, 56.4% received SNAP benefits in 11 or 12 months of the year, and 22% received TANF benefits in one or more months of the year (again, with fewer months being more common). For those who earned at least an average of $50 per month—a low threshold—most worked in all four quarters of the year, suggesting some degree of continuity with respect to earnings. Those in the Work only and Work and Welfare groups had an annual respondent income more than double that of the Welfare only group (see Table 1), suggesting that efforts to support low-income families in the workforce and facilitate the combination of work and welfare may be worthy strategies for moving up the income ladder. However, respondent incomes remain quite low, even for those who worked (i.e., less than $18,000 for the year, on average). Even if some respondents receive additional income from spouses and partners, which we were not able to account for, it is not likely that the families of working respondents are able to move out of poverty. The very fact that they remain eligible for WIC benefits suggests that escaping poverty remains elusive.

### Limitations

This study has several limitations that must be considered when interpreting the results. First, our list of available income sources is not exhaustive. We lacked information on the earnings and income contributions of spouses or partners, and other informal sources of income (e.g., from family and friends, local charities). As a result, respondent income is likely to more closely reflect household income among single caregivers than partnered caregivers, and household income is more relevant for understanding economic stress and hardship than respondent income alone. We also did not have information on receipt or amounts of the Earned Income Tax Credit (EITC), the Low-Income Heating and Energy Assistance Program (LI-HEAP), or housing subsidies—all benefits that are relatively common among low-income families with minor-aged children in the United States. (Receipt of Medicaid, a means-tested public health insurance program, was available, but nearly all sample members received this benefit, rendering it, essentially, a constant).

Second, we rely on investigated CPS reports to indicate child maltreatment. For an incident of maltreatment to be investigated by CPS, it requires that a community member became aware of the maltreatment, reported it, and provided enough information that a CPS worker deemed it appropriate to investigate. As a result, this measure likely misses many abusive or neglectful acts, particularly among families who may be in infrequent contact with mandatory reporters.

Third, the data used for this study were drawn from a survey with a low to moderate response rate, and it is possible that non-respondents differ from respondents in ways important for child maltreatment or CPS involvement. Given these survey completion ratios, the resulting sample of respondents cannot be viewed as representative of the total WIC-eligible population of primary caregivers in the targeted geographic sites. The sample does, however, offer a means for assessing, in an exploratory way, the variation in earnings and benefit packages within a group of low-income families with young children, and how these packages differentially predict CPS involvement. Related to the sample, the study included a home visiting program site in addition to WIC sites. Although non-WIC recipients from the home visiting program were not included in the study and we control for the home visiting site in the statistical models, it is still possible that the inclusion of this site biases our findings. We conducted a sensitivity test of our final model, dropping sample members from the home visiting program site, and the results are nearly identical to the final model.

The lack of findings in this analysis with respect to socioemotional measures such as parenting stress, depressive symptoms, and social support run contrary to some (but not all) of the extant literature. In stepwise logistic regression models (not shown), the depressive symptoms measure has a marginally (*p* < 0.10) statistically significant and positive association with CPS involvement that is not sensitive to the inclusion of other control variables, with the exception of housing instability. Only when housing instability is added to the model does the depressive symptoms measure become statistically insignificant (*p* < 0.18). This suggests that housing instability and depression may have a unique relationship with respect to CPS risk, which deserves additional attention. It could also be the case that participating in the WIC program in and of itself offers a protective benefit vis-à-vis these measures, as another study suggests [24], but that we are unable to test in the present study given that WIC participation is a constant. Additionally, although our socio-emotional measures demonstrate strong internal reliability, they may not adequately capture the underlying constructs.

Finally, this analysis cannot speak to causality. Individuals become engaged with work or the welfare system for reasons related to desire, need, and opportunity, all of which may independently affect child maltreatment and CPS involvement.

## 5. Conclusions

This study has several implications for policy and practice to support families and prevent maltreatment. Given the WIC program’s ability to reach a relatively large proportion of a population at a greater risk of maltreatment (i.e., families in poverty), the program has the potential to serve as an important force in the overall maltreatment prevention continuum. Understanding the full scope of a client’s economic safety net, including where there are unmet needs that could be addressed with existing resources, may be an effective means of preventing child abuse and neglect among this population. One of WIC’s current functions is making referrals to other health and social services—through additional training and resources, WIC staff members could serve as a point of assessment for other needs and facilitate connecting families to these benefits.

Findings from this exploratory analysis cautiously suggest that supporting work among low-income families may be one tool for reducing the risk of child maltreatment. However, low-wage work often fails to move a family out of poverty [46], and therefore, combining work and welfare may be necessary to the stability and well-being of the household, although being an insufficient strategy for leaving poverty behind. Given that the work-attached subgroups demonstrated higher levels of income compared to nonworking sample members, income undoubtedly represents an important mediator in the relationship between work and CPS risk. Additionally, work in the absence of sufficient ancillary supports to help parents balance the demands of employment and caregiving in the context of poverty, may have an adverse effect on family functioning, a point which deserves focused attention in future research. Finally, given the connection between housing instability and maltreatment risk, policies and programs that provide housing assistance or help families stabilize their living situation may play a critical prevention role. WIC programs could assess housing stability and work to build relationships with other community programs that assist low-income families with finding affordable housing.

## Figures and Tables

**Table 1:** Sample characteristics by work and welfare status.

Measures	Total Sample (*n* = 1065)	No Work/No Welfare (*n* = 210)	Welfare Only (*n* = 310)	Work Only (*n* = 203)	Work and Welfare (*n* = 342)
	% or mean (sd)	% or mean (sd)	% or mean (sd)	% or mean (sd)	% or mean (sd)
Post-survey CPS involvement ***	7.4%	1.9%	12.9%	2.0%	9.1%
Any prior CPS involvement ***	16.7%	10.0%	23.6%	5.9%	21.1%
Milwaukee Home visiting site ***	6.6%	9.1%	15.2%	6.9%	6.4%
Cohabiting spouse or partner ***	61.8%	73.8%	61.3%	66.0%	52.3%
High school degree (ref.) **	45.7%	43.3%	45.5%	47.8%	39.5%
<High school degree ***	16.3%	17.1%	25.5%	4.4%	14.6%
>High school degree ***	38.0%	39.5%	29.0%	47.8%	39.5%
Non-Hispanic White/Other (ref.) ***	65.4%	60.0%	55.8%	78.3%	69.6%
Non-Hispanic Black ***	10.6%	6.2%	15.8%	3.9%	12.6%
Hispanic ***	19.4%	31.4%	25.2%	10.3%	12.3%
Parent < 25 years old **	36.6%	29.5%	33.6%	39.9%	41.8%
More than 2 children in home ***	34.7%	46.2%	39.7%	22.2%	30.4%
At least one child < 2 years old ***	6.2%	4.3%	4.8%	11.8%	5.3%
Parenting stress	13.7 (3.1)	14.0 (3.3)	13.8 (2.9)	13.3 (2.9)	13.7 (3.2)
Depressive symptoms **	30.4 (10.4)	29.6 (10.5)	31.2 (10.4)	28.8 (10.0)	31.2 (10.5)
Social support ***	30.6 (5.6)	30.0 (5.3)	29.9 (5.7)	32.1 (5.1)	30.6 (5.8)
Housing instability ***	18.5%	16.2%	18.1%	11.8%	24.3%
No work and No welfare	19.7%	-	-	-	-
Welfare and No work (ref.)	29.1%	-	-	-	-
Work and No welfare	19.1%	-	-	-	-
Work and Welfare	32.1%	-	-	-	-
SSI Receipt (self or children) ***	3.7%	0.9%	10.7%	0.5%	0.9%
Total annual income ***	$11,176 ($11,296)	$935 ($2697)	$7537 ($5722)	$16,299 ($11,884)	$17,724 ($10,662)

*** *p* < 0.01; ** *p* < 0.05.

**Table 2:** Logistic regression predicting CPS involvement (*n* = 1065).

Measures	B	SE	OR
Any prior CPS involvement ***	1.93	0.283	6.90
Milwaukee Home visiting site	0.07	0.51	1.07
Cohabiting spouse or partner	−0.31	0.28	0.74
High school degree (ref.)	-	-	-
<High school degree	0.34	0.33	1.41
>High school degree *	−0.61	0.33	0.54
Non-Hispanic White or Other (ref.)	-	-	-
Hispanic	−0.60	0.46	0.55
Non-Hispanic Black	0.35	0.44	1.42
Parent < 25 years old	0.21	0.32	1.23
More than 2 children in home	0.20	0.29	1.22
At least one child < 2 years old **	0.98	0.47	2.68
Parenting stress	0.05	0.05	1.05
Depressive symptoms	0.02	0.01	1.02
Social support	−0.01	0.03	0.99
Housing instability **	0.70	0.30	2.02
Welfare only (ref.)	-	-	-
No Welfare/No work	0.18	0.67	1.19
Work only **	−1.47	0.59	0.23
Work and Welfare *	−0.61	0.33	0.54
SSI Receipt (self or children)	0.40	0.49	1.49
Total annual income (ln) ***	0.37	0.14	1.44
Constant **	−7.01	1.90	0.001

B: Beta; SE: Standard Error of Beta; OR: Odds Ratio; *** *p* < 0.01; ** *p* < 0.05; * *p* < 0.10.
